# Proteomic characterization of SMARCA-deficient esophageal adenocarcinoma reveals complement evasion and DNA repair-associated prognostic subgroups

**DOI:** 10.1038/s41416-026-03534-7

**Published:** 2026-07-08

**Authors:** Bastian Grothey, Max Krämer, Matteo Montalbano, Su Ir Lyu, Christiane J. Bruns, Thomas Zander, Reinhard Büttner, Alexander Quaas

**Affiliations:** 1 Institute of Pathology, Faculty of Medicine and University Hospital of Cologne, University of Cologne, Cologne, Germany; 2 Department I of Internal Medicine, Center for Integrated Oncology (CIO), University of Cologne, Cologne, Germany; 3 Pathology Unit, Department of Experimental Oncology, Mediterranean Institute of Oncology, Catania, Italy; 4 Department of General, Visceral and Cancer Surgery, Faculty of Medicine and University Hospital of Cologne, Cologne, Germany

**Keywords:** Predictive markers, Prognostic markers, Oesophageal cancer, Tumour immunology, Mass spectrometry

## Abstract

**Background:**

Oesophageal adenocarcinoma (EAC) is an aggressive malignancy in which the SWI/SNF catalytic subunits SMARCA2 and SMARCA4 are frequently lost, yet the biological and clinical impact of these deficiencies remains unclear.

**Methods:**

We analysed 113 EAC specimens using mass spectrometry–based proteomics, including 89 SMARCA-deficient and 24 SMARCA-proficient tumours. Additional immunohistochemical studies were conducted on 98 cases. Differential expression, pathway enrichment, and survival analyses were used to characterise proteomic alterations and identify prognostic features.

**Results:**

SMARCA-deficient tumours showed frequent expression of complement-inactivating proteins CD55 and CD59 with distinctive apical and membranous localisation. SMARCA4-deficient patients had significantly poorer survival than both SMARCA2-deficient and SMARCA-proficient patients. Among neoadjuvantly treated SMARCA-deficient tumours, coordinated upregulation of DNA repair proteins separated patients into two prognostic subgroups. The DNA repair–low subgroup had markedly worse survival than both the DNA repair–high subgroup and SMARCA-proficient controls, whereas the DNA repair–high subgroup showed a trend toward improved outcomes. This DNA repair–associated pattern was not observed in neoadjuvantly treated SMARCA-proficient tumours.

**Conclusions:**

SMARCA-deficient EAC comprises biologically distinct subgroups with prognostic proteomic signatures. CD55 and CD59 expression may contribute to resistance to antibody-based immunotherapies by complement evasion, while DNA repair profiles could guide post-surgical treatment in neoadjuvantly treated patients.

## Introduction

Oesophageal adenocarcinoma (EAC) represents a highly aggressive malignancy of the gastrointestinal tract, with rapidly rising incidence rates [1–3]. Despite advances in multimodal treatment approaches, the overall prognosis remains poor with 5-year survival rates of 20% [3]. The heterogeneous nature of EAC presents significant challenges for developing effective therapeutic strategies and identifying reliable predictive biomarkers.

The SWI/SNF (SWItch/Sucrose Non-Fermentable) chromatin remodelling complex plays a pivotal role in regulating gene expression through ATP-dependent nucleosome repositioning and chromatin accessibility [4]. This multiprotein complex assembles in distinct configurations, with SMARCA2 (BRM) and SMARCA4 (BRG1) acting as mutually exclusive catalytic ATPase subunits required for its activity [4, 5]. Genomic alterations affecting SWI/SNF components are among the most common aberrations in human cancers, with inactivating mutations or epigenetic silencing occurring in approximately 25% of malignancies [6]. These disruptions have been associated with reduced overall survival in several cancer types and may confer exploitable therapeutic vulnerabilities [7–10]. Preclinical studies indicate that SWI/SNF-deficient tumours can exhibit synthetic lethality and heightened sensitivity to targeted interventions, including PARP inhibitors and immune checkpoint blockade [7, 8]. In addition, there are now early clinical trials investigating selective SMARCA2 degraders (PROTAC-like molecules) that specifically target and degrade the remaining SWI/SNF ATPase subunit SMARCA2 (BRM) to therapeutically exploit the synthetic lethal vulnerability of SMARCA4-deficient tumours [11, 12].

Deficiencies in the SWI/SNF ATPase subunits SMARCA2 and SMARCA4 have been implicated in oncogenesis across multiple cancer types, showing high prevalence in lung, bladder, and gastrointestinal malignancies [6, 13]. In oesophageal cancer, SMARCA2 and SMARCA4 deficiencies (SMARCA-deficiency) have been reported in 9.9% and 3.4% of cases, respectively, and loss of SMARCA4 has been correlated with higher histological grade [9]. However, the functional consequences of SMARCA-deficiency in EAC remain poorly understood, particularly regarding how loss of these proteins influences tumour biology, therapeutic response, and clinical outcomes.

To advance our understanding of SMARCA-deficiency in EAC, improved molecular characterisation is critically needed. While genomic-based approaches provide valuable insights, they may not fully capture the functional impact of chromatin remodelling defects. Protein expression patterns and pathway activation states offer more direct insights into cellular phenotypes and functional consequences. However, comprehensive proteomic characterisation of SMARCA-deficient EAC tumours remains limited. Mass spectrometry-based proteomics presents a compelling opportunity to systematically characterise protein expression signatures associated with SMARCA deficiency.

In this study, we performed mass spectrometry-based proteomic analysis to characterise the proteome of SMARCA-deficient oesophageal adenocarcinoma (EAC). Our analysis revealed that SMARCA-deficient tumours frequently express the complement-inactivating proteins CD55 and CD59, with subsequent immunohistochemistry analyses demonstrating distinct expression patterns characterised by reproducible apical and membranous staining in tumour cells. Importantly, we found that SMARCA4-deficient EAC exhibited significantly inferior survival compared to both SMARCA-proficient EAC and SMARCA2-deficient EAC, highlighting distinct prognostic implications of different SMARCA subunit deficiencies. Among neoadjuvant-treated patients with SMARCA-deficient tumours showing incomplete response to therapy, DNA repair protein expression stratified cases into two distinct subgroups. Notably, tumours exhibiting coordinated upregulation of DNA repair proteins demonstrated significantly improved overall survival outcomes compared to those without such upregulation. These findings provide novel insights into the molecular heterogeneity of SMARCA-deficient EAC and establish specific protein expression signatures that may serve as prognostic biomarkers for patient stratification and guide targeted therapeutic interventions.

## Methods

### Study design and patient cohort

This retrospective study included 793 patients with adenocarcinoma of the oesophagus who underwent surgical resection at the Department of General, Visceral, Cancer, and Transplantation Surgery of the University Hospital Cologne. All data were collected prospectively and analysed retrospectively. Clinical and pathological characteristics were documented according to standard protocols, following the 7th edition of the Union for International Cancer Control. The study was conducted in accordance with the Declaration of Helsinki and approved by the Ethics Committee of the University Hospital Cologne (reference number protocol code 20-1393). Written informed consent for inclusion in the institutional databank and tissue bank was obtained from each patient prior to surgery.

**Fig. 1:**
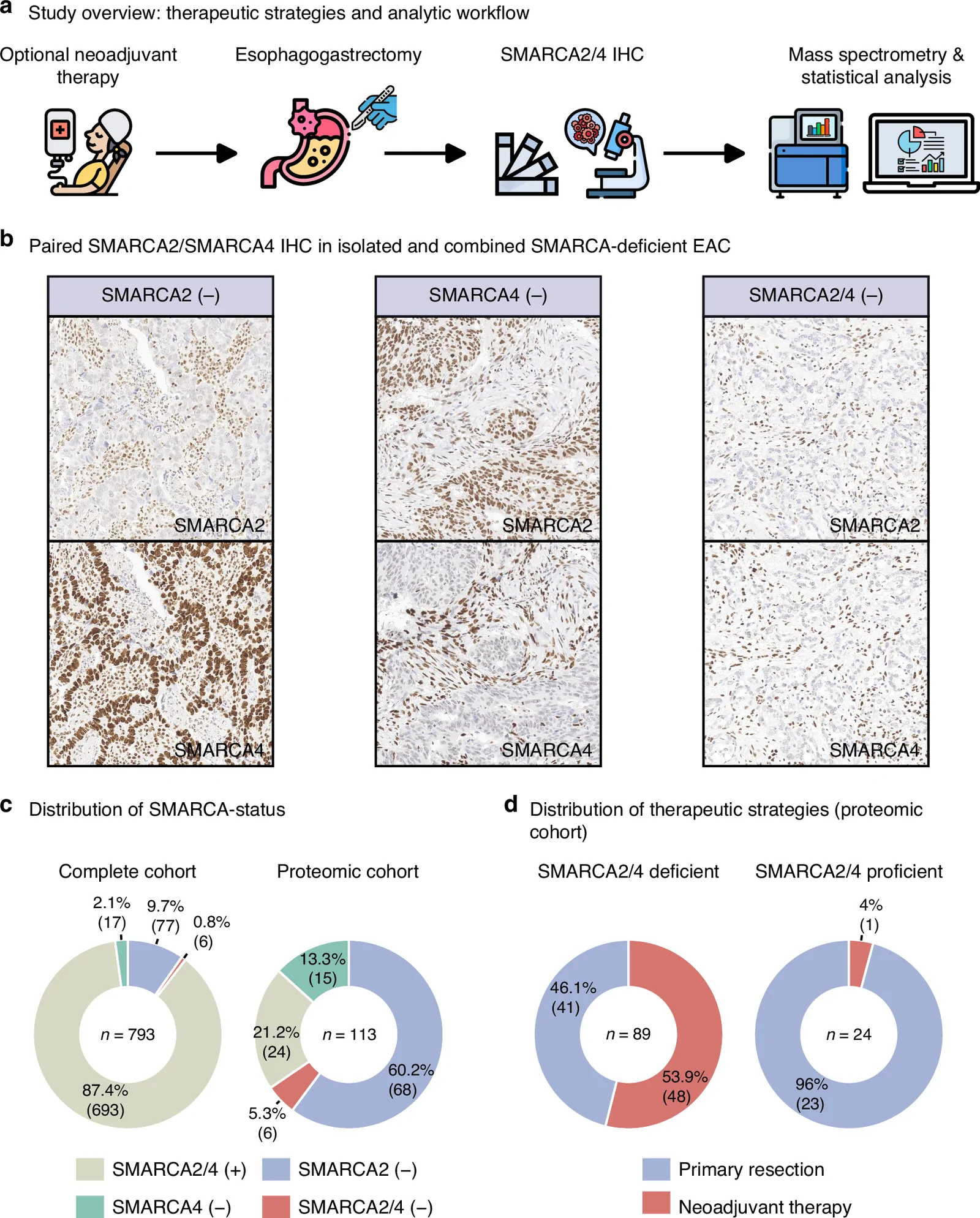
Study design and cohort composition. **a** Schematic representation of the therapeutic strategies and analytic workflow. Patients with EAC either received neoadjuvant chemotherapy/radiochemotherapy followed by esophago-gastrectomy or underwent primary esophago-gastrectomy without neoadjuvant therapy. SMARCA expression status was determined by immunohistochemistry in EAC specimens from 793 patients obtained from esophago-gastrectomy, using tissue microarrays (TMAs) for IHC screening. A cohort of 115 tumours was subsequently selected for mass spectrometry-based proteomic analysis and quantitative characterisation of protein expression. Graphical illustrations were obtained from Flaticon. **b** Representative immunohistochemical images illustrating SMARCA expression patterns in SMARCA-deficient EAC. For each deficiency subgroup, isolated SMARCA2 loss (SMARCA2-), isolated SMARCA4 loss (SMARCA4-), and combined SMARCA2/4 loss (SMARCA2/4-), paired images show SMARCA2 (top) and SMARCA4 (bottom) immunoreactivity. Complete absence of nuclear staining in tumour cells defines the respective deficient protein, while intratumoral stromal and inflammatory cells serve as internal positive controls. **c** Distribution of SMARCA expression status in the complete cohort (left) and proteomic analysis subset (right). The complete cohort of 793 EAC cases included: 77 cases with SMARCA2 loss (SMARCA2 − ), 17 cases with SMARCA4 loss (SMARCA4 − ), 6 cases with combined SMARCA2/4 loss (SMARCA2/4 − ), and 693 cases with retained SMARCA expression (SMARCA2/4 + ). The 113 tumours analysed by mass spectrometry comprised: 68 cases with SMARCA2 loss, 15 cases with SMARCA4 loss, 6 cases with combined SMARCA2/4 loss, and 24 cases with retained SMARCA expression. Percentages are indicated within each segment, with absolute patient numbers given in parentheses. **d** Distribution of therapeutic strategies stratified by SMARCA status. Among SMARCA-deficient EAC patients, 48 received neoadjuvant therapy and 41 underwent primary resection. Among SMARCA-proficient EAC patients, 1 received neoadjuvant therapy and 23 underwent primary resection. Percentages are indicated within each segment, with absolute patient numbers given in parentheses.

Immunohistochemical screening of SMARCA2 and SMARCA4 expression status was conducted across the complete cohort of 793 EAC cases obtained from esophago-gastrectomies (Fig. 1a/b). Partial data were incorporated from our group’s previous study, which had already assessed SMARCA2/4 immunohistochemical staining in a subset of these cases [9]. SMARCA-deficiency was identified in 100 cases (12.6%) of the complete cohort, with isolated SMARCA2 loss being the most frequent pattern observed in 77 cases (9.7%), followed by isolated SMARCA4 loss in 17 cases (2.1%), and combined loss of both SMARCA2 and SMARCA4 proteins in 6 cases (0.8%) (Fig. 1c). The remaining 693 cases (87.4%) retained expression of both SMARCA-proteins. We subsequently selected 115 tumour and 13 normal oesophageal tissue specimens for mass spectrometry-based proteomic analysis. SMARCA-deficient tumour cases were selected to ensure a balanced representation of patients who received neoadjuvant therapy and patients who underwent primary surgery, while SMARCA-proficient tumours were randomly selected from patients who underwent primary surgery. One case was retrospectively identified as having received neoadjuvant treatment and was reclassified accordingly in all analyses. Two of the 115 samples were identified as outliers in mass spectrometry and were excluded from further analyses. The final cohort comprised 113 samples for proteomic analysis, consisting of 68 cases (60.2%) with SMARCA2 loss, 15 cases (13.3%) with SMARCA4 loss, 6 cases (5.3%) with combined SMARCA2/4 loss, and 24 cases (21.2%) with retained SMARCA expression (Fig. 1c). Among the 89 patients with SMARCA-deficient tumours, 48 patients (53.9%) received neoadjuvant therapy prior to surgical resection, while 41 patients (46.1%) underwent primary resection without neoadjuvant treatment (Fig. 1d). In contrast, among the 24 patients with SMARCA-proficient tumours, only 1 patient (4.2%) received neoadjuvant therapy, with the vast majority (23 patients, 95.8%) proceeding directly to primary resection. Additional patient characteristics are summarised in Table 1. Further proteomic data from 20 neoadjuvant-treated SMARCA-proficient patients were obtained from a previous research project conducted by our group, as previously described [14]. For immunohistochemical validation studies of CD55 expression, we analysed 98 EAC samples from patients who underwent primary surgical resection, comprising 37 SMARCA-deficient tumours and 61 SMARCA-proficient tumours.

**Table 1:** Clinicopathological characteristics of patients included in the proteomic analysis cohort.

	Total		SMARCA-Status		
Parameter		Deficient		Proficient	Fisher’s exact test
	*n* (%)	*n* (%)		*n* (%)	*p*-value
No. of patients	113	89		24	
Gender					0.5478
Female	19 (16.8)	14 (15.7)		5 (20.8)	
Male	94 (83.2)	75 (84.3)		19 (79.2)	
Age					0.3343
Age ( < 65)	46 (40.7)	39 (43.8)		7 (29.2)	
Age (>=65)	61 (54)	46 (51.7)		15 (62.5)	
Unknown	6 (5.3)	4 (4.5)		2 (8.3)	
(y)pT					0.3129
0	0 (0)	0 (0)		0 (0)	
1	14 (12.4)	9 (10.1)		5 (20.8)	
2	22 (19.5)	19 (21.3)		3 (12.5)	
3	71 (62.8)	57 (64)		14 (58.3)	
4	6 (5.3)	4 (4.5)		2 (8.3)	
(y)pN					0.6739
0	37 (32.7)	29 (32.6)		8 (33.3)	
1	38 (33.6)	32 (36)		6 (25)	
2	18 (15.9)	13 (14.6)		5 (20.8)	
3	20 (17.7)	15 (16.9)		5 (20.8)	
L					**0.0479**
0	42 (37.2)	34 (38.2)		8 (33.3)	
1	37 (32.7)	22 (24.7)		15 (62.5)	
Unknown	34 (30.1)	33 (37.1)		1 (4.2)	
V					0.5744
0	80 (70.8)	58 (65.2)		22 (91.7)	
1	4 (3.5)	2 (2.2)		2 (8.3)	
Unknown	29 (25.7)	29 (32.6)		0 (0)	
Pn					1.0000
0	20 (17.7)	11 (12.4)		9 (37.5)	
1	25 (22.1)	14 (15.7)		11 (45.8)	
Unknown	68 (60.2)	64 (71.9)		4 (16.7)	

The table presents baseline clinicopathological characteristics of 113 oesophageal adenocarcinoma patients selected for mass spectrometry-based proteomic analysis, stratified by SMARCA expression status (SMARCA-deficient vs. SMARCA-proficient). Fisher’s exact test was used to assess statistical significance between groups. Significant differences were observed only in lymphovascular invasion (*p* < 0.05). Bold print marks *p*-value below 0.05.

*pT* Histopathological tumour stage, *pN* Histopathological nodal status, *L* Lymphovascular invasion, *V* Vascular invasion, *Pn* Perineural invasion.

### Tissue microarray construction and immunohistochemistry

For tissue microarray (TMA) construction, tumour tissue specimens were formalin-fixed and paraffin-embedded (FFPE) according to standard histopathological protocols. Representative tumour areas were identified by experienced pathologists. Tissue cylinders with a diameter of 1.2 mm were punched from selected tumour regions using a semi-automated precision instrument and embedded in recipient paraffin blocks to create TMAs. Immunohistochemical staining was performed using the automated staining system Leica BOND-MAX (Leica Biosystems, Wetzlar, Germany). Detailed information regarding antibodies is provided in Supplementary Table S1.

SMARCA2/4 expression status was screened across the complete cohort using single-spot TMAs. For immunohistochemical validation studies of CD55 expression, SMARCA-deficient tumours were analysed using a heterogeneity TMA established in our research laboratory, consisting exclusively of SMARCA-deficient cases with 4 different tumour spots per case. SMARCA-proficient tumours were evaluated on single spot TMAs.

Immunohistochemical scoring was performed using the H-Score system, calculated as follows: H-Score = 1 × (% cells with weak staining) + 2 × (% cells with moderate staining) + 3 × (% cells with strong staining), yielding scores ranging from 0 to 300 [15, 16]. The following H-Score thresholds were applied: 0-50 (negative), 51-100 (weak positive), 101-200 (moderate positive), and 201-300 (strong positive). Apical and membranous staining patterns were scored separately. For cases analysed on the heterogeneity TMA of SMARCA-deficient tumours, H-Scores were calculated as the mean across all four tumour spots per case. All scoring was performed by trained pathologists.

### Protein extraction and mass spectrometry analysis

Protein extraction from FFPE tissue sections and subsequent mass spectrometry analysis were performed according to established protocols. A detailed protocol for sample preparation, mass spectrometry parameters, data processing, and outlier definition is provided in the Supplementary Methods. Proteomic data from the additional cohort of 20 neoadjuvant-treated SMARCA-proficient patients, obtained from a previous study by our group, were processed following the protocol described in the respective primary publication [14].

### Differential expression analysis and pathway enrichment

Differential protein expression analysis was performed using the limma package in R [17]. Proteins with *p *< 0.05 and adjusted *p* < 0.2 were considered significantly differentially expressed. Pathway enrichment analysis was conducted using both KEGG and Reactome databases implemented through the clusterProfiler and ReactomePA R packages, respectively [18, 19]. Overrepresentation analysis was performed using two distinct significance thresholds: differentially expressed proteins were selected using *p* < 0.05 adjusted *p* < 0.2, while survival-associated proteins were selected using *p* < 0.05 without adjustment for multiple comparisons.

### Oncogene and tumour suppressor gene annotation

Annotation of oncogenes and tumour suppressor genes used in survival analyses were obtained from the OncoKB database (https://www.oncokb.org/).

### Statistical analysis and data visualisation

All statistical analyses and data visualisations were performed using R (version 4.4.2, R Foundation for Statistical Computing, Vienna, Austria). Graphical illustrations were created using Inkscape (https://inkscape.org/), and additional illustrations were obtained from Flaticon (https://www.flaticon.com; permission for publication obtained).

### Disclosure of artificial intelligence assistance

Claude 4 Sonnet (Anthropic, https://claude.ai) was used via the desktop application for language refinements, spelling and grammar corrections of the manuscript, and coding support during data analysis.

## Results

### SMARCA-deficiency EAC frequently exhibit expression complement-inactivating proteins CD55 and CD59

**Fig. 2:**
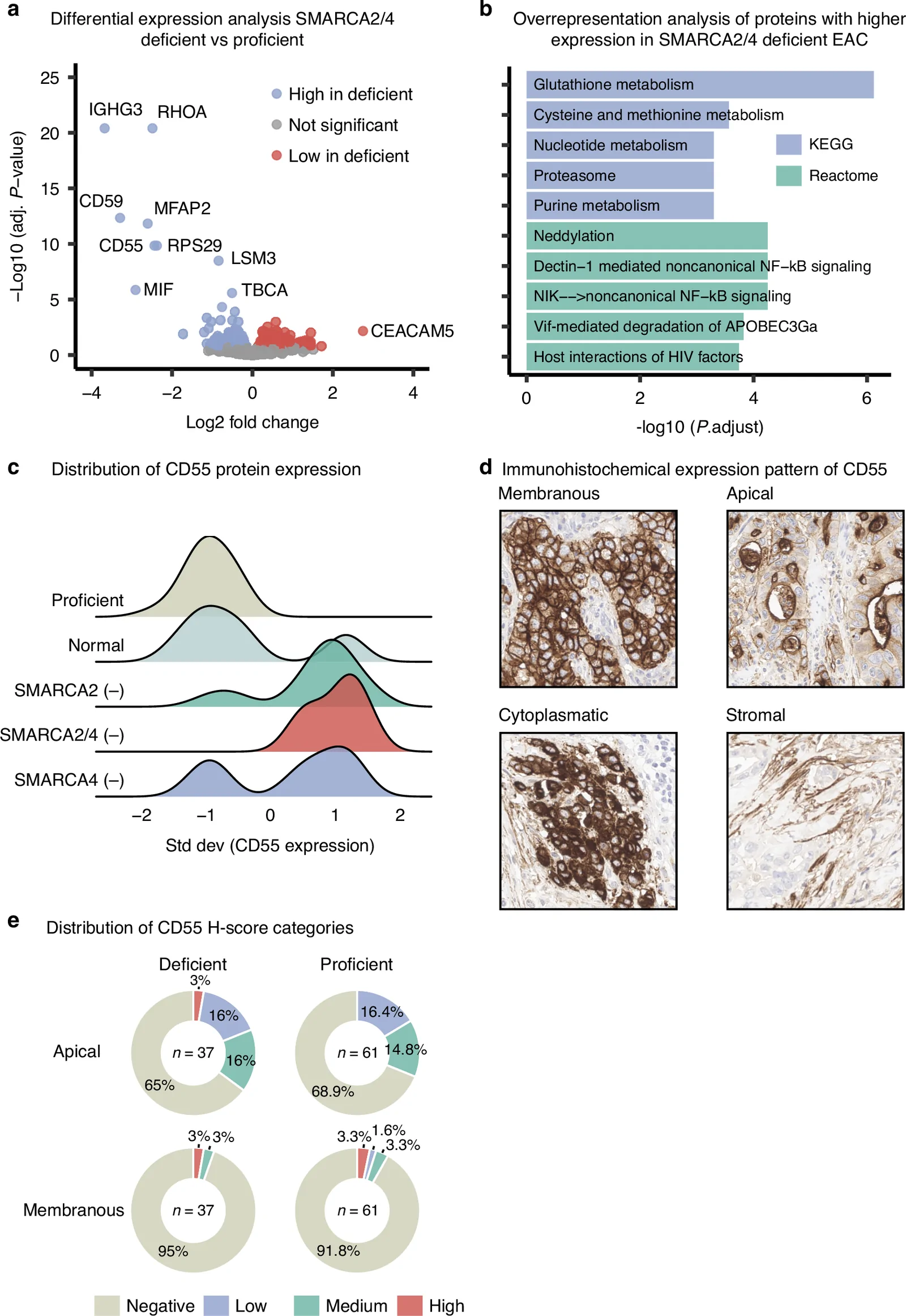
Proteomic characterisation of SMARCA-deficient EAC from primary surgery. **a** Volcano plot of differential protein expression between SMARCA-deficient and -proficient EAC from primary surgery specimens (*n* = 64). Of 4324 quantified proteins, 655 are differentially expressed (*p* < 0.05, adjusted *p* < 0.2). Blue dots indicate proteins with higher expression in SMARCA-deficient tumours (negative log2 fold change), red dots indicate proteins with lower expression in deficient tumours (positive log2 fold change), and grey dots represent non-significant changes. Selected proteins are labelled. **b** Pathway enrichment (overrepresentation) analysis of proteins upregulated in SMARCA-deficient EAC using KEGG (blue) and Reactome (green) databases. Pathways are ranked by significance (-log10 adjusted *p*-value), showing enrichment in metabolic processes (glutathione, cysteine/methionine, and nucleotide metabolism), neddylation, and noncanonical NF-κB signalling pathways. For each database top 5 significantly enriched pathways are shown. **c** Distribution of CD55 protein expression (standardised) across SMARCA-deficiency subgroups and normal tissue determined by mass spectrometry (*n* = 73). SMARCA-deficient tumours show elevated CD55 expression compared to proficient tumours and normal tissue. While proficient and combined-deficient SMARCA2/4 tumours exhibit unimodal distributions, single-deficient tumours (SMARCA2 or SMARCA4 alone) display more heterogeneous, bimodal expression patterns. Proficient = SMARCA-proficient, Normal = Normal tissue, SMARCA2 (−) = SMARCA2 deficient, SMARCA4 (−) = SMARCA4 deficient, SMARCA2/4 (−) = combined SMARCA2/4 deficiency. **d** Representative immunohistochemical staining patterns of CD55: membranous, apical, cytoplasmic, and stromal localisation. **e** CD55 H-score distribution determined by immunohistochemistry in SMARCA-proficient (*n* = 61) and SMARCA-deficient (*n* = 37) EAC tumours, presented as categorical data. No significant differences were observed in either apical or membranous CD55 expression between SMARCA-deficient and SMARCA-proficient tumours (apical: *p* = 0.71; membranous: *p* = 1.0; Fisher’s exact test).

Following mass spectrometry-based proteomic analysis, we performed differential expression analysis comparing SMARCA-deficient and -proficient EAC specimens from primary surgery (*n* = 64 patients without neoadjuvant therapy) to identify protein signatures associated with SMARCA-deficiency. Mass spectrometry analysis quantified 4324 proteins, of which 655 demonstrated differential expression between SMARCA-deficient and -proficient tumours (*p* < 0.05, adjusted *p* < 0.2) (Fig. 2a; Supplementary Table S2). Proteins with higher expression in SMARCA-deficient tumours included complement-inactivating proteins (CD55, CD59), inflammatory mediators (MIF), signal transduction (RHOA) and extracellular matrix components (MFAP2), while proteins with lower expression included carcinoembryonic antigen-related cell adhesion molecules (CEACAM5). Pathway enrichment analysis of upregulated proteins in SMARCA-deficient tumours revealed significant enrichment in metabolic pathways, particularly glutathione metabolism, cysteine and methionine metabolism, and nucleotide metabolism (Fig. 2b; Supplementary Tables S3–S4). Additionally, protein neddylation and noncanonical NF-κB signalling pathways, including NIK-mediated and Dectin-1 mediated signalling, were significantly enriched.

Among the differentially expressed proteins identified by mass spectrometry, CD55 (complement decay-accelerating factor) and CD59 (membrane attack complex inhibition factor) were among the most robustly upregulated proteins in SMARCA-deficient tumours. Both proteins were also upregulated when comparing SMARCA-deficient tumours to normal oesophageal tissue (Supplementary Table S5).

Mass spectrometry analysis revealed elevated CD55 expression across all SMARCA-deficient subgroups compared to SMARCA-proficient tumours and normal oesophageal tissue (Fig. 2c). Notably, while SMARCA-proficient and combined-deficient (SMARCA2/4⁻) tumours exhibited unimodal CD55 expression distributions, single-deficient tumours (SMARCA2⁻ or SMARCA4⁻ alone) displayed more heterogeneous, bimodal expression patterns.

To identify the specific cell populations expressing CD55 and to validate the proteomic findings, we performed immunohistochemical analysis using tissue microarrays. We analysed 98 EAC samples total: 37 SMARCA-deficient tumours and 61 SMARCA-proficient tumours. Immunostaining revealed CD55 expression by tumour cells with four distinct subcellular localisation patterns: membranous, apical, cytoplasmic, and stromal, which occasionally coexisted within individual tissue sections (Fig. 2d). Since cytoplasmic and stromal staining occurred infrequently, we focused subsequent comparative analyses on membranous and apical staining patterns on the tumour cells. Interestingly, in contrast to the mass spectrometry analysis, H-score analysis showed no statistically significant differences in CD55 expression between SMARCA-deficient and SMARCA-proficient tumours using either categorical analysis (apical: *p* = 0.71; membranous: *p* = 1.0; Fisher’s exact test) (Fig. 2e) or quantitative comparison (apical: *p* = 0.076; membranous: *p* = 0.41; Wilcoxon test).

### Increased expression of ERBB2 and DNAJB1 is associated with poor prognosis in primary resected SMARCA-deficient EAC

**Fig. 3:**
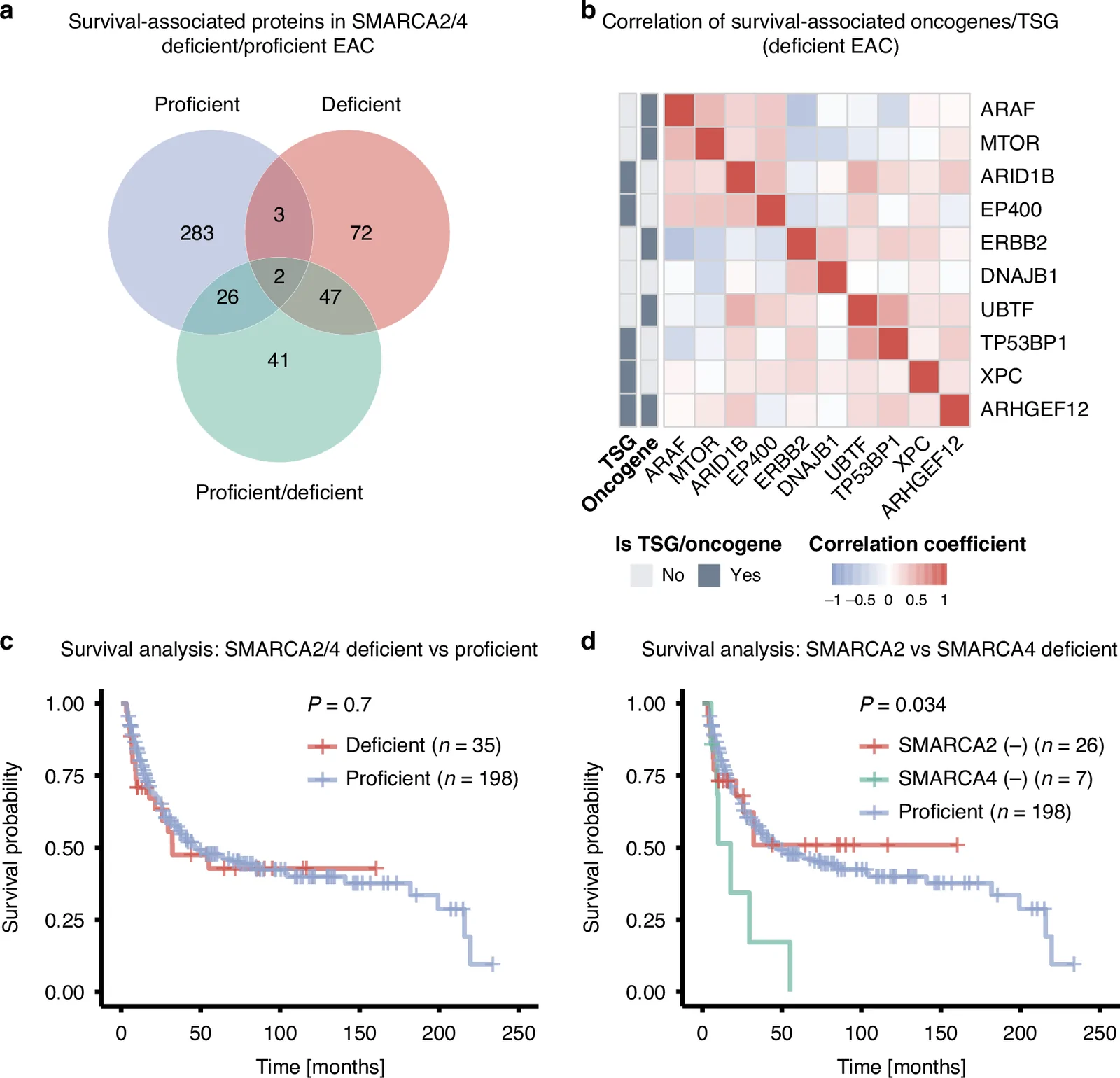
Survival-associated protein expression patterns and clinical outcomes in primary resected SMARCA-deficient. **a** Venn diagram showing the distribution of survival-associated proteins identified through univariable Cox proportional hazards regression analysis in SMARCA-deficient EAC (*n* = 30), SMARCA-proficient EAC (*n* = 15), and the combined cohort (*n* = 45). Numbers represent proteins significantly associated with overall survival (*p* < 0.05, unadjusted for multiple comparisons). **b** Correlation heatmap displaying pairwise Pearson correlation coefficients for survival-associated oncogenes and tumour suppressor genes (TSGs) within the SMARCA-deficient EAC. Colour intensity indicates correlation strength (scale: -1 to +1), with red representing positive correlations and blue representing negative correlations. Grey and black sidebar annotations indicate TSG/oncogene classification. **c** Kaplan–Meier survival analysis comparing overall survival between SMARCA-deficient (red line, *n* = 35) and SMARCA-proficient (blue line, *n* = 198) EAC patients. No statistically significant difference was observed between groups (*p* = 0.7, log-rank test). **d** Kaplan-Meier analysis stratified by SMARCA subunit deficiency: SMARCA2-deficient (red, *n* = 26), SMARCA4-deficient (green, *n* = 7), and SMARCA-proficient (blue, *n* = 198) patients. SMARCA4-deficient patients demonstrated significantly worse overall survival compared to both SMARCA2-deficient and SMARCA-proficient groups, while no significant difference existed between SMARCA2-deficient and SMARCA-proficient patients (overall log-rank test *p* = 0.034; pairwise comparisons: SMARCA-proficient vs. SMARCA2-deficient *p* = 0.8633, SMARCA-proficient vs. SMARCA4-deficient *p* = 0.0086, SMARCA2-deficient vs. SMARCA4-deficient *p* = 0.0452).

Next, we performed univariable Cox proportional hazards regression analysis to identify survival-associated proteins in primary surgery specimens. Among SMARCA-deficient tumours, 124 proteins demonstrated significant associations (*p* < 0.05) with overall survival (*n* = 30), whereas 314 proteins showed survival associations in SMARCA-proficient tumours (*n* = 15) (Fig. 3a; Supplementary Table S6). Given the large number of protein expressions tested, we did not apply multiple testing corrections. When filtering for oncogenes and tumour suppressor genes annotated in OncoKB, we identified 10 different proteins associated with survival in patients with SMARCA-deficient tumours (Fig. 3b). These include proteins associated with DNA repair (TP53BP1, XPC, EP400) [20–23], chromatin remodelling (ARID1B—a SWI/SNF complex component—and EP400) [24, 25], and growth signalling (ARAF, ERBB2, MTOR) [26, 27]. Correlation analysis of protein expression levels revealed moderate positive correlations among ARAF, MTOR, ARID1B, and EP400, while ARAF and ERBB2 exhibited moderate to strong negative correlation. Regarding prognostic impact, elevated expression of ERBB2 and DNAJB1 was associated with increased hazard ratios (poorer survival), whereas higher expression of the remaining proteins correlated with decreased hazard ratios (improved survival).

Having identified these associations between protein expression and survival outcomes in SMARCA-deficient EAC, we examined whether general differences in survival existed among the SMARCA-defined patient subgroups in our cohort. Kaplan–Meier survival analysis showed no significant difference in overall survival between SMARCA-deficient and -proficient patients (*p* = 0.7, log-rank test) (Fig. 3c). However, when stratifying by specific SMARCA subunit deficiency (SMARCA2 vs SMARCA4 deficiency), SMARCA4-deficient patients showed significantly poorer overall survival compared to both SMARCA2-deficient and SMARCA-proficient patients (*p* = 0.034, overall log-rank test) (Fig. 3d). In pairwise comparisons SMARCA4-deficient patients had significantly worse survival than SMARCA-proficient patients (*p* = 0.0086, log-rank test) and SMARCA2-deficient patients (*p* = 0.0452, log-rank test), while no significant difference was observed between SMARCA2-deficient and SMARCA-proficient patients (*p* = 0.8633, log-rank test).

### Coordinated DNA repair pathway activation associates with improved outcomes in neoadjuvant-treated SMARCA-deficient EAC

Current clinical practice guidelines recommend neoadjuvant therapy for patients with EAC, including those harbouring SMARCA-deficient tumours [28]. Tumours subjected to neoadjuvant treatment may experience selective pressure during therapy, potentially altering their oncogenic driver mechanisms compared to treatment-naive, primarily resected specimens. These molecular differences may have significant implications for the development of targeted therapeutic strategies and predictive biomarkers. To address this possibility, we analysed neoadjuvant-pretreated patients with SMARCA-deficient tumours to identify associations between protein expression patterns and survival outcomes.

**Fig. 4:**
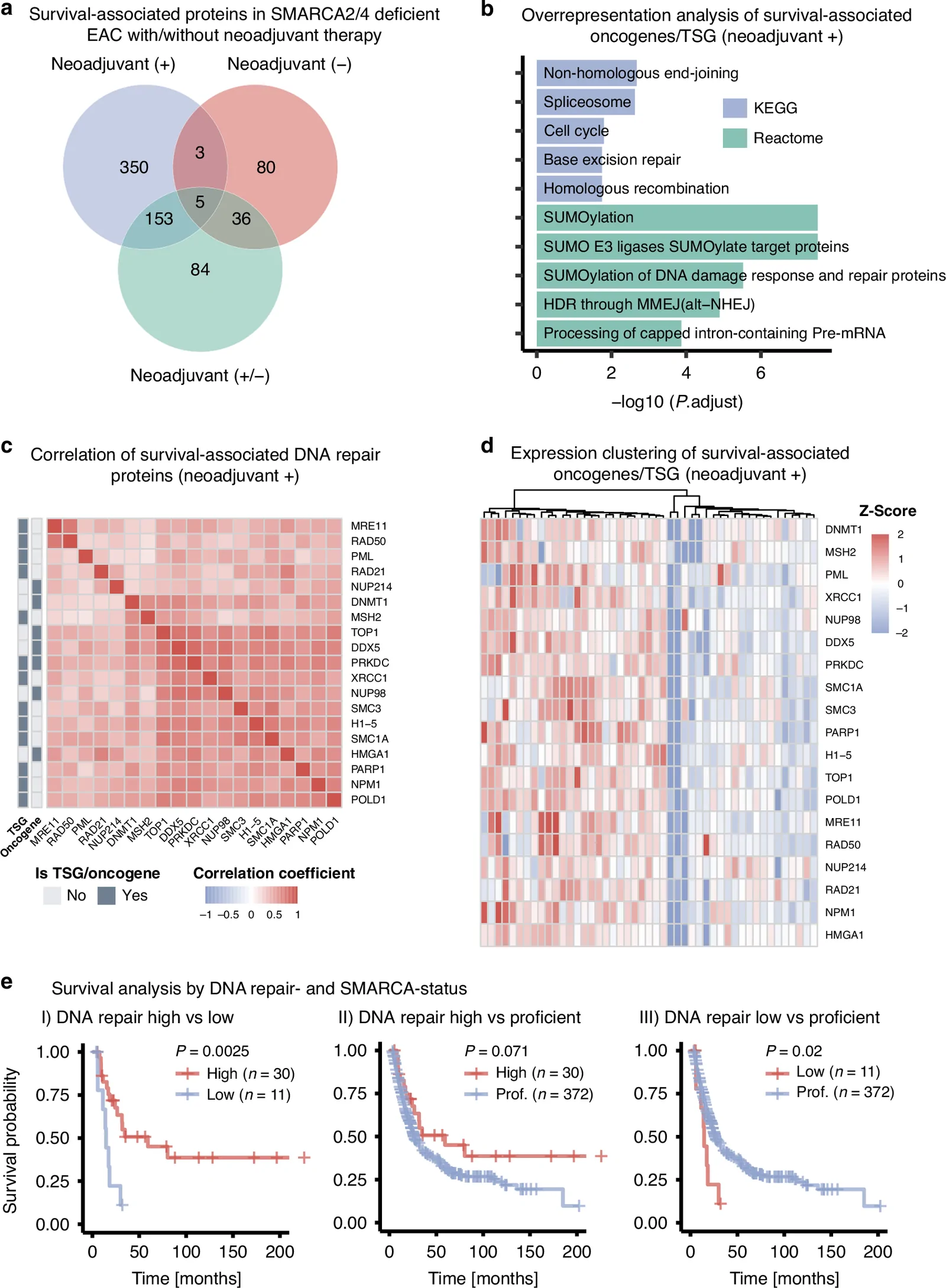
Survival-associated protein expression patterns and clinical outcomes in neoadjuvant-treated SMARCA-deficient EAC patients. **a** Venn diagram depicting overlap of survival-associated proteins identified by univariable Cox regression analysis in SMARCA-deficient EAC patients receiving neoadjuvant therapy (*n* = 41), without neoadjuvant therapy (*n* = 30), and combined analysis (*n* = 71). Numbers represent proteins significantly associated with overall survival (*p* < 0.05, unadjusted for multiple comparisons). **b** Pathway enrichment (overrepresentation) analysis of survival-associated oncogenes and tumour suppressor genes (TSGs) in SMARCA-deficient, neoadjuvant-treated EAC patients, showing top 5 significantly enriched KEGG (blue) and Reactome (green) pathways (adjusted *p* < 0.05). DNA damage response and repair pathways, including homologous recombination, non-homologous end-joining, base excision repair, and SUMOylation processes, are prominently represented. **c** Correlation matrix of survival-associated DNA repair proteins in SMARCA-deficient, neoadjuvant-treated EAC patients. Colour intensity indicates Pearson correlation coefficients (scale: −1 to +1). Grey and black sidebar annotations indicate TSG/oncogene classification. Strong positive correlations are observed among key DNA repair proteins. **d** Expression heatmap of survival-associated DNA repair proteins in SMARCA-deficient, neoadjuvant-treated EAC patients. Colour intensity represents standardised expression Z-scores (scale: -2 to +2). Unsupervised clustering identifies two distinct subgroups characterised by high versus low expression of DNA repair-associated proteins. **e** Kaplan–Meier survival analysis comparing neoadjuvant-treated SMARCA-deficient patients stratified by DNA repair expression (High, *n* = 30; Low, *n* = 11) versus neoadjuvant-treated SMARCA-proficient controls (Prof., *n* = 372). Patient stratification utilised k-means clustering based on survival- and DNA repair-associated proteins. The DNA repair-low subgroup exhibits significantly inferior survival compared to both DNA repair-high (*p* = 0.0025) and SMARCA-proficient groups (*p* = 0.02). DNA repair-high patients show a trend toward superior survival versus SMARCA-proficient patients (*p* = 0.071; all *p*-values by log-rank test).

We performed separate univariable Cox regression analyses within the SMARCA-deficient cohort, stratifying patients by neoadjuvant therapy status. Among patients with SMARCA-deficient tumours who received neoadjuvant therapy (*n* = 41), univariable Cox regression analysis identified 511 proteins significantly associated with overall survival (*p* < 0.05, unadjusted for multiple comparisons), compared to 124 proteins in patients who underwent primary resection of SMARCA-deficient tumours without neoadjuvant treatment (*n* = 30) (Fig. 4a; Supplementary Table S7). To assess whether this difference could be attributable to an unequal distribution of SMARCA deficiency subtypes between the two treatment groups, we applied Fisher’s exact test comparing the composition of SMARCA2- versus SMARCA4-deficient cases between neoadjuvant-treated and treatment-naïve patients. No significant difference in subtype composition was detected (*p* = 0.6563), indicating that treatment group assignment is not confounded by differential SMARCA subtype distribution.

When focusing on oncogenes and tumour suppressor genes within the neoadjuvant-treated SMARCA-deficient cohort, pathway enrichment analysis revealed an enrichment in DNA damage response and repair pathways (Fig. 4b; Supplementary Table S8/9). The most significantly enriched pathways included homologous recombination, non-homologous end-joining, base excision repair, and SUMOylation processes involved in DNA damage response. Additional enriched pathways encompassed cell cycle regulation and spliceosome function. Correlation analysis of survival-associated DNA repair proteins in neoadjuvant-treated patients demonstrated strong positive correlations among key components of DNA damage response pathways, including PMS2, MRE11, RAD50, PML, RAD21, and other proteins involved in homologous recombination and DNA repair mechanisms (median correlation coefficient 0.52) (Fig. 4c).

To determine whether the observed DNA repair pathway enrichment represents a characteristic specific to SMARCA-deficient tumours or a general feature of EAC following neoadjuvant therapy, we analysed an independent cohort of SMARCA-proficient EAC patients. Due to the limited number of SMARCA-proficient patients who received neoadjuvant therapy in our primary cohort, we reanalysed mass spectrometry data from a separate cohort previously studied in a different project. This validation analysis included 20 SMARCA-proficient EAC patients who received neoadjuvant therapy. Univariable Cox regression analyses identified 325 proteins significantly associated with survival (*p* < 0.05, without multiple testing correction) (Supplementary Table S10). Subsequent overrepresentation analysis of survival-associated proteins annotated as oncogenes or tumour suppressor genes in the OncoKB database revealed enrichment of proteins associated with signal transduction by growth receptors, among other pathways (Supplementary Table S11/12). Notably, however, no enrichment of DNA repair-associated proteins was observed in this SMARCA-proficient cohort.

The results from the SMARCA-deficient cohort indicate coordinated alterations of DNA repair protein expression (e.g., PMS2, MRE11, RAD50, PML, RAD21) in patients who received neoadjuvant treatment. To identify expression patterns and potential subgroups within these tumours, we performed unsupervised clustering of survival-associated DNA repair proteins in neoadjuvant-treated SMARCA-deficient patients. This analysis revealed two distinct expression subgroups: one characterised by high expression of DNA repair-associated proteins and another by low expression (Fig. 4d). K-means clustering identified 33 patients with high DNA repair protein expression and 15 patients with low expression profiles. To confirm that these subgroups do not merely reflect differences in SMARCA deficiency subtype composition, Fisher’s exact test was applied comparing the distribution of SMARCA2- versus SMARCA4-deficient cases between the DNA repair-high and DNA repair-low groups. No significant difference was observed (*p* = 0.3492), supporting that the identified subgroups represent biologically distinct entities independent of SMARCA subtype. Kaplan–Meier survival analysis of these subgroups revealed significant prognostic differences (Fig. 4e). Patients in the DNA repair-high subgroup exhibited significantly superior overall survival compared to the DNA repair-low subgroup (*p* = 0.0025, log-rank test). To contextualise these findings, we compared both subgroups against neoadjuvant-treated SMARCA-proficient patients (*n* = 372) as a reference cohort. The DNA repair-high subgroup exhibited a trend to improved overall survival relative to SMARCA-proficient patients (*p* = 0.071, log-rank test), while the DNA repair-low subgroup demonstrated significantly inferior survival outcomes compared to this reference group (*p* = 0.02, log-rank test).

## Discussion

Oesophageal adenocarcinoma remains one of the most challenging malignancies to treat, with heterogeneous tumour biology contributing to poor clinical outcomes and limited therapeutic options [1–3]. While genomic profiling has provided valuable insights into the mutational landscape of EAC [29], protein expression patterns may offer more direct functional readouts of cellular phenotypes and pathway activation states.

This study represents the first comprehensive mass spectrometry-based proteomic characterisation of SMARCA-deficient EAC. Our proteomic analysis of 113 EAC specimens has uncovered previously unrecognised molecular features of SMARCA-deficient tumours, including frequent expression of complement-inactivating proteins (CD55 and CD59) and treatment-specific DNA repair pathway activation. These findings advance our understanding of the functional consequences of chromatin remodelling defects in EAC and establish protein expression signatures that may serve as prognostic biomarkers and guide therapeutic decision-making.

An important finding of our proteomic analysis was the frequent expression of complement-inactivating proteins CD55 and CD59 across all SMARCA-deficient subgroups. Both proteins serve as membrane-bound inhibitors of complement activation, with CD55 accelerating the decay of C3 and C5 convertases and CD59 preventing formation of the membrane attack complex [30]. The distinct apical and membranous expression patterns we observed through immunohistochemical stainings may suggest strategic positioning to maximise complement inhibition at critical cellular interfaces.

Mass spectrometry analysis revealed significantly elevated CD55 expression in SMARCA-deficient tumours relative to both SMARCA-proficient tumours and normal tissue controls. However, immunohistochemical validation in an expanded patient cohort failed to demonstrate significant differences in CD55 expression between SMARCA-deficient and SMARCA-proficient tumours, regardless of staining pattern (apical or membranous). This apparent discordance between proteomic and immunohistochemical results likely reflects inherent methodological differences between these complementary analytical approaches. Mass spectrometry provides quantitative protein measurements with high sensitivity and dynamic range, potentially detecting subtle expression changes below the threshold of visual discrimination in semi-quantitative immunohistochemical analysis [31, 32]. Furthermore, the larger immunohistochemical validation cohort included additional samples not represented in the initial mass spectrometry dataset, providing broader population representation that may have contextualised our initial proteomic observations.

Regardless of these results, upregulated CD55/CD59 expression has been documented across diverse tumour types, indicating that complement evasion is not exclusively associated with SMARCA-deficiency but rather represents a broader oncogenic mechanism [33–37]. Notably, to our knowledge, CD55/CD59 expression has not been previously reported in EAC. Our findings suggest that complement-inactivating protein expression constitutes a general feature of EAC biology, potentially reflecting selective pressure for immune evasion during tumorigenesis. However, further investigations are needed to determine whether clinically relevant differences between SMARCA-proficient and SMARCA-deficient EAC exist that may be detectable by the superior sensitivity of mass spectrometry but fall below the threshold of immunohistochemical detection.

Upregulation of CD55 and CD59 in EAC has important implications for immune evasion mechanisms and tumour microenvironment dynamics. By preventing complement-dependent cytotoxicity, these proteins enable tumour cells to evade complement-mediated immune surveillance, potentially contributing to immune privilege within the tumour microenvironment [30]. This protective mechanism parallels findings in HER2-positive breast cancer, where CD55 and CD59 expression has been associated with resistance to trastuzumab therapy, which relies partially on complement-dependent cytotoxicity for its anti-tumour effects [36].

These findings are particularly relevant in the context of emerging targeted therapies for gastroesophageal cancers. For example, Trastuzumab (anti-HER2) and Zolbetuximab (anti-Claudin18.2) are monoclonal antibodies approved for treating oesophageal and gastric cancer that depend partially on complement activation for their therapeutic efficacy [36, 38]. The frequent expression of CD55 and CD59 in SMARCA-deficient EAC may represent a mechanism which can lead to resistance to complement-dependent therapies, potentially limiting the effectiveness of these antibodies and similar agents in this patient subset. Consequently, CD55 expression could serve as a predictive biomarker for Trastuzumab/Zolbetuximab resistance, while also identifying tumours that might benefit from combination approaches targeting both complement-inactivating proteins and their primary therapeutic targets.

Our analysis of treatment-naive SMARCA-deficient EAC revealed several potential compensatory mechanisms that influence patient outcomes. The association between higher EP400 expression and improved survival suggests a critical role for chromatin remodelling backup mechanisms that may prevent more aggressive phenotypes. EP400, the catalytic subunit of the TIP60 complex, provides alternative ATP-dependent chromatin remodelling activity that may compensate for lost SWI/SNF function [25]. This compensation mechanism is supported by literature demonstrating that EP400-containing complexes can regulate many of the same genomic loci targeted by SWI/SNF complexes, suggesting functional redundancy that may preserve essential chromatin remodelling activities in SMARCA-deficient tumours [25].

Similarly, the survival benefit associated with ARID1B upregulation reflects within-complex compensation mechanisms. As a SWI/SNF complex component, ARID1B upregulation may help maintain residual complex function despite SMARCA2/4 loss, potentially preserving critical gene regulatory programs [39]. This finding suggests that SMARCA-deficient tumours with retained expression of other SWI/SNF components may be less functionally compromised than those with complete complex disruption, creating opportunities for synthetic lethality approaches that target remaining chromatin remodelling activities.

Conversely, elevated expression of ERBB2 and DNAJB1 was associated with poorer survival outcomes, indicating that activation of growth signalling pathways and protein folding stress responses may promote more aggressive tumour behaviour in SMARCA-deficient EAC. These proteins represent potential therapeutic targets that could improve outcomes in high-risk patient subsets.

Overall survival did not differ significantly between SMARCA-deficient and SMARCA-proficient treatment-naive patients. However, subunit-specific analysis revealed significant survival differences. SMARCA4-deficient patients demonstrated significantly worse overall survival compared to both SMARCA2-deficient and SMARCA-proficient patients, while SMARCA2-deficient patients showed survival outcomes comparable to SMARCA-proficient controls. These findings suggest distinct prognostic implications and potential functional differences between SMARCA2 and SMARCA4 ATPase subunits in oesophageal adenocarcinoma.

The analysis of neoadjuvant-treated SMARCA-deficient EAC revealed a markedly different survival-associated protein landscape compared to treatment-naive tumours, characterised by prominent enrichment of DNA repair pathways including homologous recombination, non-homologous end-joining, and base excision repair (e.g., PMS2, MRE11, RAD50, PML, RAD21). Expression levels of DNA repair proteins consistently correlated with improved survival outcomes and stratified neoadjuvant-treated SMARCA-deficient tumours into two distinct subgroups: DNA repair-high and DNA repair-low. Patients in the DNA repair-high subgroup demonstrated superior survival compared to the DNA repair-low group. The strong positive correlations observed among these DNA repair proteins indicate coordinated upregulation of damage response mechanisms in the DNA repair-high subgroup, reflecting a systematic cellular response to therapy-induced genomic stress rather than isolated protein changes. Importantly, no similar enrichment pattern was observed in a separate limited cohort of neoadjuvant-treated SMARCA-proficient EAC patients, indicating some degree of specificity for SMARCA-deficient tumours.

These findings indicate that SMARCA-deficiency may promote either vulnerabilities or resistance mechanisms to DNA-damaging therapies which are absent in tumours with intact chromatin remodelling function. Two potential mechanisms may account for this differential therapeutic response. First, SMARCA-deficiency could promote cellular plasticity, thereby conferring enhanced resistance to DNA-damaging agents [40]. This hypothesis is supported by the significantly worse survival observed in SMARCA-deficient patients within the DNA repair-low subgroup compared to their SMARCA-proficient counterparts. Alternatively, given the well-established role of SWI/SNF complexes in DNA damage repair processes, SMARCA-loss may directly compromise repair mechanisms by impairing both damage recognition and repair efficiency [41]. Consistent with this model, SMARCA-deficient patients in the DNA repair-high subgroup showed a trend toward improved survival compared to SMARCA-proficient patients. This therapeutic vulnerability is corroborated by experimental evidence showing that SMARCA-deficient tumours exhibit sensitivity to PARP and CDK9 inhibitors in both in vitro and in vivo models [42]. Such repair impairment could trigger compensatory upregulation of alternative DNA repair pathways, representing an adaptive cellular response to persistent genomic instability.

Irrespective of the underlying mechanism, these findings may facilitate identification of patients who would benefit from continued FLOT chemotherapy following neoadjuvant therapy and surgery, or alternatively, those who require alternative treatment strategies, thereby minimising unnecessary toxicity while optimising therapeutic outcomes.

Some limitations of our study warrant acknowledgement. The limited number of SMARCA-proficient patients who received neoadjuvant therapy restricted our ability to fully characterise treatment-dependent changes in this subgroup, necessitating the use of an independent validation cohort. Additionally, our analysis focused on protein expression patterns rather than functional validation of the proposed mechanisms, highlighting the need for complementary in vitro and in vivo studies to confirm the functional significance of CD55/CD59 expression and DNA repair pathway activation.

Future research aspects include assessment of CD55/CD59 inhibition as a therapeutic strategy, and prospective clinical validation of CD55/CD59 expression as predictive biomarkers for Claudin18.2-targeted therapy. Additionally, further preclinical and clinical studies are needed to determine whether DNA repair protein expression can reliably identify SMARCA-deficient EAC patients who would benefit from continued FLOT chemotherapy following neoadjuvant therapy and surgery, versus those who might respond better to alternative treatment approaches. Furthermore, the enrichment of IGHG3 in SMARCA2/4-deficient EAC alongside the frequent expression of complement-inactivating proteins CD55 and CD59 raises intriguing questions about the interplay between humoral immune responses and complement evasion in these tumours. A comprehensive characterisation of the immune-related tumour microenvironment in SMARCA-deficient EAC represents an important direction for future investigation.

## Conclusions

Our proteomic analysis of SMARCA-deficient oesophageal adenocarcinoma has revealed novel insights into tumour biology, immune evasion mechanisms, and treatment-dependent molecular reprogramming. The identification of CD55 and CD59 expression in SMARCA-deficient EAC provides a mechanistic basis for potential therapeutic resistance and highlights opportunities for biomarker development and combination therapeutic strategies. Furthermore, the coordinated DNA repair pathway activation in neoadjuvant-treated SMARCA-deficient tumours represents an important finding that could inform post-surgical therapeutic decisions. These results establish a foundation for several future research directions: functional validation of our proteomic findings, prospective clinical validation of identified biomarkers, and development of targeted therapeutic strategies in SMARCA-deficient tumours.

## Supplementary information

Supplementary tables

Supplementary methods

## Data Availability

The mass spectrometry proteomics data have been deposited to the ProteomeXchange Consortium via the PRIDE partner repository with the dataset identifier PXD074975 [43, 44]. Preprocessed mass spectrometry data and clinicopathological annotations are publicly available on Zenodo (https://doi.org/10.5281/zenodo.18823589). Further data that support the findings of this study are available from the corresponding authors upon request.

## Code availability

Source code for data analysis performed on the preprocessed data is available on Zenodo (https://doi.org/10.5281/zenodo.18823589).

## References

[1] Sung H, Ferlay J, Siegel RL, Laversanne M, Soerjomataram I, Jemal A, et al. Global cancer statistics 2020: GLOBOCAN estimates of incidence and mortality worldwide for 36 cancers in 185 countries. CA Cancer J Clin. 2021;71:209–49. 10.3322/caac.21660. 33538338

[2] Siegel RL, Miller KD, Jemal A. Cancer statistics, 2019. CA Cancer J Clin. 2019;69:7–34. 10.3322/caac.21551. 30620402

[3] Pennathur A, Gibson MK, Jobe BA, Luketich JD. Oesophageal carcinoma. Lancet. 2013;381:400–12. 10.1016/S0140-6736(12)60643-6. 23374478

[4] Wilson BG, Roberts CWM. SWI/SNF nucleosome remodellers and cancer. Nat Rev Cancer. 2011;11:481–92. 10.1038/nrc3068. 21654818

[5] Phelan ML, Sif S, Narlikar GJ, Kingston RE. Reconstitution of a core chromatin remodeling complex from SWI/SNF subunits. Mol Cell. 1999;3:247–53. 10.1016/S1097-2765(00)80315-9. 10078207

[6] Mittal P, Roberts CWM. The SWI/SNF complex in cancer—biology, biomarkers and therapy. Nat Rev Clin Oncol. 2020;17:435–48. 10.1038/s41571-020-0357-3. 32303701 PMC8723792

[7] Shen J, Peng Y, Wei L, Zhang W, Yang L, Lan L, et al. ARID1A deficiency impairs the DNA damage checkpoint and sensitizes cells to PARP inhibitors. Cancer Discov. 2015;5:752–67. 10.1158/2159-8290.CD-14-0849. 26069190 PMC4497871

[8] Shen J, Ju Z, Zhao W, Wang L, Peng Y, Ge Z, et al. ARID1A deficiency promotes mutability and potentiates therapeutic antitumor immunity unleashed by immune checkpoint blockade. Nat Med. 2018;24:556–62. 10.1038/s41591-018-0012-z. 29736026 PMC6076433

[9] Schallenberg S, Bork J, Essakly A, Alakus H, Buettner R, Hillmer AM, et al. Loss of the SWI/SNF-ATPase subunit members SMARCF1 (ARID1A), SMARCA2 (BRM), SMARCA4 (BRG1) and SMARCB1 (INI1) in oesophageal adenocarcinoma. BMC Cancer. 2020;20:12. 10.1186/s12885-019-6425-3. 31906887 PMC6945480

[10] Savas S, Skardasi G. The SWI/SNF complex subunit genes: their functions, variations, and links to risk and survival outcomes in human cancers. Crit Rev Oncol Hematol. 2018;123:114–31. 10.1016/j.critrevonc.2018.01.009. 29482773

[11] Cantley J, Ye X, Rousseau E, Januario T, Hamman BD, Rose CM, et al. Selective PROTAC-mediated degradation of SMARCA2 is efficacious in SMARCA4 mutant cancers. Nat Commun. 2022;13:6814. 10.1038/s41467-022-34562-5. 36357397 PMC9649729

[12] Hulse M, Wang M, Xu C, Carter J, Agarwal A, Lu L, et al. PRT3789 is a first-in-human SMARCA2-selective degrader that induces synthetic lethality in SMARCA4-mutated cancers. Cancer Res. 2025. 10.1158/0008-5472.CAN-25-1141. 40991405

[13] Reisman D, Glaros S, Thompson EA. The SWI/SNF complex and cancer. Oncogene. 2009;28:1653–68. 10.1038/onc.2009.4. 19234488

[14] Grothey B, Lyu SI, Quaas A, Simon AG, Jung JO, Schröder W, et al. Proteomic characterization of MET-amplified esophageal adenocarcinomas reveals enrichment of alternative splicing- and androgen signaling-related proteins. Cell Mol Life Sci. 2025;82:112 10.1007/s00018-025-05635-7. 40074836 PMC11904063

[15] Goulding H, Pinder S, Cannon P, Pearson D, Nicholson R, Snead D, et al. A new immunohistochemical antibody for the assessment of estrogen receptor status on routine formalin-fixed tissue samples. Hum Pathol. 1995;26:291–4. 10.1016/0046-8177(95)90060-8. 7890280

[16] McCarty KS, Miller LS, Cox EB, Konrath J, McCarty KS. Estrogen receptor analyses. Correlation of biochemical and immunohistochemical methods using monoclonal antireceptor antibodies. Arch Pathol Lab Med. 1985;109:716–21. 3893381

[17] Ritchie ME, Phipson B, Wu D, Hu Y, Law CW, Shi W, et al. limma powers differential expression analyses for RNA-sequencing and microarray studies. Nucleic Acids Res. 2015;43:e47–e47. 10.1093/nar/gkv007. 25605792 PMC4402510

[18] Guangchuang YU, Aut C clusterProfiler [Internet]. Bioconductor; 2017 [cited 2025 Sep 27]. Available from: https://bioconductor.org/packages/clusterProfiler. .

[19] Yu G, He QY. ReactomePA: an R/Bioconductor package for Reactome pathway analysis and visualization. Mol Biosyst. 2016;12:477–9. 10.1039/C5MB00663E. 26661513

[20] Rappold I, Iwabuchi K, Date T, Chen J. Tumor suppressor P53 binding protein 1 (53bp1) is involved in DNA damage–signaling pathways. J Cell Biol. 2001;153:613–20. 10.1083/jcb.153.3.613. 11331310 PMC2190566

[21] Mirza-Aghazadeh-Attari M, Mohammadzadeh A, Yousefi B, Mihanfar A, Karimian A, Majidinia M. 53BP1: a key player of DNA damage response with critical functions in cancer. DNA Repair. 2019;73:110–9. 10.1016/j.dnarep.2018.11.008. 30497961

[22] Boulikas T. Xeroderma pigmentosum and molecular cloning of DNA repair genes. Anticancer Res. 1996;16:693–708. 8687116

[23] Lu PYT, Lévesque N, Kobor MS. NuA4 and SWR1-C: two chromatin-modifying complexes with overlapping functions and components. Biochem Cell Biol. 2009;87:799–815. 10.1139/O09-062. 19898529

[24] Odnokoz O, Banerjee A, Cui X, Zeng L, Uddin A, Li C, et al. Disruption of ARID1B recruitment to the nuclear pore complex as a new anticancer therapeutic strategy. Adv Sci. 2025;12:e15585. 10.1002/advs.202415585. PMC12463049 40671262

[25] Martin BJE, Ablondi EF, Goglia C, Mimoso CA, Espinel-Cabrera PR, Adelman K. Global identification of SWI/SNF targets reveals compensation by EP400. Cell. 2023;186:5290–5307.e26. 10.1016/j.cell.2023.10.006. 37922899 PMC11307202

[26] Ullah R, Yin Q, Snell AH, Wan L. RAF-MEK-ERK pathway in cancer evolution and treatment. Semin Cancer Biol. 2022;85:123–54. 10.1016/j.semcancer.2021.05.010. 33992782

[27] Huang XL, Khan MI, Wang J, Ali R, Ali SW, Zahra QulA, et al. Role of receptor tyrosine kinases mediated signal transduction pathways in tumor growth and angiogenesis—new insight and futuristic vision. Int J Biol Macromol. 2021;180:739–52. 10.1016/j.ijbiomac.2021.03.075. 33737188

[28] Shah MA, Kennedy EB, Catenacci DV, Deighton DC, Goodman KA, Malhotra NK, et al. Treatment of locally advanced esophageal carcinoma: ASCO guideline. J Clin Oncol. 2020;38:2677–94. 10.1200/JCO.20.00866. 32568633

[29] The Cancer Genome Atlas Research NetworkIntegrated genomic characterization of oesophageal carcinoma. Nature. 2017;541:169–75. 10.1038/nature20805. 28052061 PMC5651175

[30] Geller A, Yan J. The role of membrane bound complement regulatory proteins in tumor development and cancer immunotherapy. Front Immunol. 2019;10:1074 10.3389/fimmu.2019.01074. 31164885 PMC6536589

[31] Gillette MA, Carr SA. Quantitative analysis of peptides and proteins in biomedicine by targeted mass spectrometry. Nat Methods. 2013;10:28–34. 10.1038/nmeth.2309. 23269374 PMC3943160

[32] Steiner C, Ducret A, Tille J, Thomas M, McKee TA, Rubbia-Brandt L, et al. Applications of mass spectrometry for quantitative protein analysis in formalin-fixed paraffin-embedded tissues. Proteomics. 2014;14:441–51. 10.1002/pmic.201300311. 24339433 PMC4265304

[33] Kesselring R, Thiel A, Pries R, Fichtner-Feigl S, Brunner S, Seidel P, et al. The complement receptors CD46, CD55 and CD59 are regulated by the tumour microenvironment of head and neck cancer to facilitate escape of complement attack. Eur J Cancer. 2014;50:2152–61. 10.1016/j.ejca.2014.05.005. 24915776

[34] Simpson KL, Jones A, Norman S, Holmes CH. Expression of the complement regulatory proteins decay accelerating factor (DAF, CD55), membrane cofactor protein (MCP, CD46) and CD59 in the normal human uterine cervix and in premalignant and malignant cervical disease. Am J Pathol. 1997;151:1455–67. 9358772 PMC1858073

[35] Patel B, Silwal A, Eltokhy MA, Gaikwad S, Curcic M, Patel J, et al. Deciphering CD59: unveiling its role in immune microenvironment and prognostic significance. Cancers. 2024;16:3699 10.3390/cancers16213699. 39518137 PMC11545456

[36] Wang Y, Yang YJ, Wang Z, Liao J, Liu M, Zhong XR, et al. CD55 and CD59 expression protects HER2-overexpressing breast cancer cells from trastuzumab-induced complement-dependent cytotoxicity. Oncol Lett. 2017;14:2961–9. 10.3892/ol.2017.6555. 28928834 PMC5588148

[37] Shao F, Gao Y, Wang W, He H, Xiao L, Geng X, et al. Silencing EGFR-upregulated expression of CD55 and CD59 activates the complement system and sensitizes lung cancer to checkpoint blockade. Nat Cancer. 2022;3:1192–210. 10.1038/s43018-022-00444-4. 36271172

[38] Shah MA, Shitara K, Ajani JA, Bang YJ, Enzinger P, Ilson D, et al. Zolbetuximab plus CAPOX in CLDN18.2-positive gastric or gastroesophageal junction adenocarcinoma: the randomized, phase 3 GLOW trial. Nat Med. 2023;29:2133–41. 10.1038/s41591-023-02465-7. 37524953 PMC10427418

[39] Hore P, Sarkar S, Bashyam MD. Evaluating the ‘ARID’ landscape of SWI/SNF complexes with relevance to cancer. Gene. 2025;965:149664. 10.1016/j.gene.2025.149664. 40652994

[40] Yuan S, Norgard RJ, Stanger BZ. Cellular plasticity in cancer. Cancer Discov. 2019;9:837–51. 10.1158/2159-8290.CD-19-0015. 30992279 PMC6606363

[41] Ribeiro-Silva C, Vermeulen W, Lans H. SWI/SNF: complex complexes in genome stability and cancer. DNA Repair. 2019;77:87–95. 10.1016/j.dnarep.2019.03.007. 30897376

[42] Zhu X, Fu Z, Aceto G, St-Germain J, Liu K, Arabzadeh A, et al. SMARCA4 loss increases RNA polymerase II pausing and elevates R-loops to inhibit BRCA1-mediated repair in ovarian cancer. Cancer Res. 2025;85:2997–3014. 10.1158/0008-5472.CAN-24-3990. 40366633

[43] Deutsch EW, Bandeira N, Perez-Riverol Y, Sharma V, Carver JJ, Mendoza L, et al. The ProteomeXchange consortium in 2026: making proteomics data FAIR. Nucleic Acids Res. 2026;54:D459–69. 10.1093/nar/gkaf1146. 41206473 PMC12807779

[44] Perez-Riverol Y, Bandla C, Kundu DJ, Kamatchinathan S, Bai J, Hewapathirana S, et al. The PRIDE database at 20 years: 2025 update. Nucleic Acids Res. 2025;53:D543–53. 10.1093/nar/gkae1011. 39494541 PMC11701690

