# Supplementary material for: Proteomic characterization of SMARCA-deficient esophageal adenocarcinoma reveals complement evasion and DNA repair-associated prognostic subgroups
Source: Br J Cancer. 2026 Jul 8;135(7):1119–30. doi: 10.1038/s41416-026-03534-7 (PMC13578737; doi:10.1038/s41416-026-03534-7)
Supplement: Supplementary file 1 [file 41416_2026_3534_MOESM2_ESM.docx]

1. **FFPE-Tissue Lysis and digestion**

FFPE tissue scrolls (roughly 100mm2 x 5 µm) were transferred to 100 µl tissue lysis buffer (TLB, Covaris) in an AFA-TUBE TPX 96 well Plate (Covaris). After sealing with strip caps, the plates were incubated at 95°C for 10 min in a thermocycler. For subsequent deparaffinization plates were transferred to a Covaris LE220-plus Focused-ultrasonicator and processed with 450 W peak power, 50% duty cycle and 200 cycles at 20°C for 5 min. To de-crosslink proteins, the samples were subsequently incubated at 95° for 90 min in a thermocycler and proteins were finally extracted by a second ultrasonication step in the Covaris LE220 using the settings described above. TCEP and chloroacetamide were added to final concentrations of 5 mM and 20 mM respectively to reduced disulfide bonds and alkylate cysteines. Samples were incubated for 20 min at 55°C in a Thermocycler. 50 µl of protein lysates were used for protein digestion following a modifies version of the single-pot solid phase enhanced sample preparation protocol (Hughes 2019). Hydrophilic and hydrophobic Sera-Mag carboxylate-modified magnetic particles (GE Healthcare) were combined 1:1, washed two times with 10 volumes of water and stored at 10 µg/µl. 2 µl of the bead mix were added to the samples and acetonitrile (ACN) was added to a final percentage of 50%. The mixture was incubated at 25°C for 8 min with shaking at 1400 rpm. The plates were placed on a magnetic rack for 2 min, supernatants were removed and the beads were washed with 180 µl prewarmed isopropanol at 55°C and 1400 rpm for 10 min. Supernatants were removed on a magnetic rack and the isopropanol washes were repeated four more times. Finally, the trapped beads were washed with 200 µl acetonitrile on the magnetic rack, air dried and reconstituted in 50 µl 50mM Triethylammonium bicarbonate (TEAB) containing 1 µg Trypsin. Proteins were digested for 16 h at 37°C in a Thermocycler. After digestion samples were acidified with 50 µl 5 % formic acid. Beads were trapped on a magnetic rack for 3 min and 90 µl supernatant containing the digested proteins were transferred to fresh plates. 5 µl of each sample were diluted with 15 ul 0.1 % formic acid and loaded on Evotips (Evosep) according to the manufacturer instructions.

2. **Mass spectrometry**

   Samples were analyzed on a timsTOF Pro2 mass spectrometer (Bruker) that was equipped with a captive spray ion source and a fused silica emitter (20 µm inner diameter, Bruker) coupled with an Evosep One LC system. The predefined 30SPD method was used with a Performance 15 cm × 150 μm C-18 column (Evosep) that was maintained at 40°C in a column oven (Bruker). The buffer system consisted of 0.1 % formic acid in water (buffer A) and B 0.1 % formic acid in acetonitrile (buffer B). MS data were acquired in dia-PASEF mode. The method uses 16 scans with variable m/z windows and 2 ion mobility windows per scan to cover a mass range from 350 m/z to 1250 m/z and an ion mobility range from 1/K0 = 0.75 V/cm2to 1/KO = 1.4 V/cm2. Scan times were 100 ms, the resulting cycle time was 1.6 sec. The ion mobility was calibrated at the start of each run using the in-batch calibration option.
3. **Data processing**

   A predicted spectral library was generated using a human canonical Swissprot fasta and DIA-NN 1.8.1 (Demichev 2020) using the following settings: Trypsin, up to 1 missed cleavage, precursor ion range 350 m/z – 1250 m/z, fragment ion range 200 m/z – 1700 m/z, charge states 2+, 3+ and 4+. Sample files were searched using this library in match between run (MBR) mode with default settings.
4. **Data Analysis**

Identifier and quantitative columns were extracted from the main DIA-NN output file and filtered. Normalised precursor quantities were used for MaxLFQ protein quantification in the DIANN R-package. Sample runs with precursor ID numbers above or below the upper or lower quartile +/- inter quartile range (4 out of 128) were regarded as outliers and removed. The protein LFQ intensities were imported into Perseus 1.6.15.0 and filtered for at least 50% data completeness. Missing values were imputed with random values from the lower end of the intensity distribution using Perseus defaults.

**Literature**

Hughes, C.S., Moggridge, S., Müller, T. *et al.* Single-pot, solid-phase-enhanced sample preparation for proteomics experiments. *Nat Protoc* **14,** 68–85 (2019).

<https://doi.org/10.1038/s41596-018-0082-x>

Demichev, V., Messner, C.B., Vernardis, S.I. *et al.* DIA-NN: neural networks and interference correction enable deep proteome coverage in high throughput. *Nat Methods* **17,** 41–44 (2020). <https://doi.org/10.1038/s41592-019-0638-x>
